# Phytogenic Compounds Supplemented to Gestating Hyperprolific Sows Affects the Gut Health-Related Gene Expression and Histological Responses in Neonate Piglets

**DOI:** 10.3389/fvets.2021.639719

**Published:** 2021-06-14

**Authors:** David Reyes-Camacho, José Francisco Pérez, Ester Vinyeta, Tobias Aumiller, Lourdes Criado-Mesas, Josep Maria Folch, Jan Dirk Van der Klis, David Solà-Oriol

**Affiliations:** ^1^Animal Nutrition and Welfare Service, Department of Animal and Food Sciences, Universitat Autònoma de Barcelona, Bellaterra, Spain; ^2^Delacon Biotechnik GmbH, Engerwitzdorf, Austria; ^3^Department of Animal Genomics, Centre for Research in Agricultural Genomics, Universitat Autònoma de Barcelona, Bellaterra, Spain

**Keywords:** hyperprolific sows, prenatal exposure, neonatal programming, phytogenic compounds, piglet gut health

## Abstract

This research aims to determine whether a specific blend of phytogenic compounds (BPC) supplemented in gestating hyperprolific sow diets can promote prenatal maternal effects in terms of piglet gut function and morphology. Twenty-eight (Landrace × Yorkshire) gilts and sows (parity 0 to 7) were randomly distributed by parity number and body weight into two dietary treatments: unsupplemented Control (CON) (*n* = 14) or CON diet supplemented with 1 g/kg feed of BPC during gestation (*n* = 14). The BPC supplementation during gestation of sows downregulated the neonate piglets' jejunal genes involved in oxidation (*SOD2*) and nutrient transport (*SLC16A1/MCT1, SLC11A2/DMT1*, and *SLC39A/ZIP4*), while *IFNG* and *CLDN4* related to immune response and barrier function, respectively, were upregulated (*q* < 0.10). In addition, the jejunal villus height and the ratio of the villus height to crypt depth tended to increase (*p* < 0.10), while goblet cell volume density was higher (*p* < 0.05) in BPC compared to CON. In conclusion, dietary supplementation of BPC in gestating diets for hyperprolific sows influences neonatal histomorphology and expression of genes related to the intestinal function and health.

## Introduction

In modern pig breeds, hyperprolific dam-line sows are characterized by a high number of fetuses *in utero* compared to conventional sow lines. This has increased the litter size; however, it has also increased the percentage of piglets affected with varying degrees of intrauterine growth retardation (IUGR) and a low birth weight (LBW) (1). The IUGR characteristics may be derived from a reduced utero-placental blood flow (2), as well as increased oxidative damage, decreased mitochondrial function, impaired angiogenesis, and downregulated protein levels of glucose transporters in placentae for LBW piglets (3). Indeed, limitations in available nutrients supplied by the sow may slow the growth of fetuses and their intestinal development (4), which are the major causes of morbidity and impaired growth performance in LBW piglets (5). Perinatal (fetal/neonatal) gut dysfunction in newborn mammals not only restricts their primary function of nutrient digestion and absorption but also could compromise their epithelial barrier function and the activation and maturation of the submucosal immune system (6). In fact, among the frequent problems suffered by IUGR piglets are intestinal growth and morphology as well as altered gene expression (7).

In previous research, we and other authors have shown that phytogenic compounds (PCs) in the sow's feed can be transferred to the offspring through amniotic fluid and milk (8, 9). This prenatal and postnatal exposure to PCs, through nutritional programming during the perinatal period, influences the oxidative status of sows and piglets (9) and also reduces weaning-associated health and welfare problems in piglets (8, 9). However, we were not able to identify the importance and relevance of early effects promoted by the prenatal dietary treatments to counteract signs of impaired gastrointestinal function in neonatal piglets. In the present study, we explored whether a blend of PCs (BPC) supplemented to the diets of gestating hyperprolific sows influences the small-intestine histomorphology and gene expression in neonate piglets. It was hypothesized that BPC supplementation would enhance the parameters related to gut health and function in neonate piglets.

## Materials and Methods

### Experimental Design, Animals, and Housing

Twenty-eight DanBred hybrid line (Landrace × Yorkshire) gilts and sows (parity 0 to 7) were randomly distributed by parity number and body weight (BW) into two dietary treatment groups (*n* = 14 per treatment). After breeding, sows were fed an unsupplemented control diet (CON) or the CON diet supplemented with 1 kg/MT of a blend of PCs (Delacon Biotechnik GmbH, Engerwitzdorf, Austria) during the entire gestation (BPC). During the entire gestation, the sows were individually controlled, while pen was included as a random effect. Sows were assigned to individual cages (1.8 × 0.8 m) from weaning (d 0) until confirmed gestation (day 35). On day 35, sows were allotted by parity and BW into group pens of seven sows/pen (5.0 × 5.0 m) until day 110 of gestation. Thereafter, sows were moved to the farrowing unit, where they were placed in individual farrowing pens (2.0 × 2.6 m) mounted over a partially slatted floor with a heated floor pad for piglets. Water was provided *ad libitum* through a nipple waterer, and experimental diets were provided to sows in pelleted form. In addition, the sows' reproductive performance was monitored at farrowing.

### Diets and Feeding

The Control diet offered in gestation was formulated to meet or exceed nutrient requirements for DanBred sows (10), with adaptations based on Spanish recommendations for gestating sows (11). The BCP experimental diet was the CON diet plus 1 g/kg of BPC supplement. The BPC contained 45 g/kg essential oils (EO) (EO composition described in Table 1). Based on individual body condition, sows were fed their corresponding diet as follows: 2.1 kg/d from weaning to service, an average of 2.9 kg/d from service to day 35 of gestation, and 2.5 kg/d from day 35 to day 110 of gestation (flat line). Each gestating pen was equipped with a mechanical free-access self-closing semi-cage without pneumatic actuators (Rotecna, Lleida, Spain) to keep animals individually monitored during feeding. From day 110 of gestation, sows were fed their corresponding treatment diet with *ad libitum* intake.

**Table 1:** Composition of supplemented EO in the BPC^a^.

**Component**	**g/kg in premix**
trans-Anethole	12.17
1.8-Cineole	9.73
Camphor	7.42
p-Cymene	2.56
D-Limonene	2.31
α-Terpineol	2.07
Borneol	1.83
α-Pinene	1.70
Linalool	1.46
β-Pinene	1.34

a *Total EO content was about 45 g/MT of feed with the 1 kg/MT premix dosage*.

### Sampling

Eight piglets per treatment (one piglet per liter, the animal representing the median BW within each litter) were selected and euthanized at farrowing (without sucking colostrum) to obtain jejunum tissue samples. Before euthanasia, piglets were anesthetized by intramuscular injection of 100 mg Telazol, 50 mg ketamine, and 50 mg xylazine per 1 ml/22 kg of BW. Thereafter, pigs were euthanized by jugular vein injection using sodium pentobarbital 0.5 ml/kg of BW. The jejunum tissue obtained for the gene expression analysis (~1.5 cm^2^) was placed in 1 ml of RNAlater (Applied Biosystems, Foster City, CA, USA) and stored at room temperature (25°C) during the first 24 h after collection. Thereafter, the tissue samples were stored at −80°C until analysis. Additional jejunum tissue samples (~2 cm^2^) were placed in individual tubes containing formaldehyde solution for histomorphology determination.

### Jejunal Gene Expression Study by qPCR

Piglet jejunum gene expression of 56 genes related to intestinal health and functionality was quantified by RT-qPCR using an OpenArray Real-Time PCR Platform as specified by Reyes-Camacho et al. (9). Briefly, total RNA was extracted from 50 mg of jejunum tissue using the Ambion RiboPure Kit (Life Technologies, Carlsbad, CA, USA) by following the manufacturer's protocol. RNA concentration was measured using a NanoDrop ND-1000 spectrophotometer (NanoDrop products, Wilmington, DE, USA), and RNA quality was checked using Agilent 2100 Bioanalyzer equipment (Agilent Technologies, Santa Clara, CA, USA). Primers were designed by spanning exon–exon boundaries using the Primer Express 2.0 software (Applied Biosystems), and genomic DNA amplification and primer dimer formation were controlled. One replicate per sample was run in a TaqMan OpenArray gene expression custom plate format for gene expression with 56 assays of 48 samples per plate (OpenArray plate) in a QuantStudio 12K Flex Real-Time PCR System (Applied Biosystems, Foster City, CA). A total of 56 genes were previously selected based on the bibliography, including four reference genes. Details regarding genes and primers can be found in previously published work by González-Solé et al. (12).

### Jejunal Histomorphology Measurement

For the histomorphology evaluation, jejunum samples were fixed for 24–48 h in neutral-buffered 10% formalin. After dehydration and embedding in paraffin wax, sections of ~3 μm were stained with hematoxylin and eosin. Villus height (VH), crypt depth (CD), number of intraepithelial lymphocyte (IEL), and number of goblet cells (GC) per 100 micrometers of villus height were measured blinded in 10 well-oriented villi and crypts per sample by the same person using a light microscope (BHS, Olympus, Barcelona, Spain).

### Statistical Analyses

The individual newborn piglet was considered as the experimental unit. All data were analyzed considering the treatment as the main effect. The results are presented as means with their corresponding SEM. For morphology, data were analyzed by using the TTEST procedure of the statistical package SAS 9.4 (SAS Inst. Inc., Cary, NC, USA), and significant differences were declared at *p* < 0.05, while 0.05 < *p* < 0.10 were considered significant tendencies.

Gene expression statistical analysis was performed as specified by González-Solé et al. (12). Briefly, data were collected and analyzed using the Thermo Fisher Cloud software 1.0 (Applied Biosystems) applying the applying the 2^−ΔΔCt^ method for relative quantification (RQ). The normal distribution of the RQ values was checked with the Shapiro.test function of R 3.5.3 software, and log2 transformation was applied if required. One-way ANOVA was performed, and the Benjamini–Hochberg false discovery rate (FDR) was used for multiple-testing correction of *p*-values, defining the model:

$${\text{Y}}_{\text{ij}}=\mu_{\text{i}}+{\text{treat}}_{\text{j}}+\;\varepsilon_{\text{ij}}$$

where Y_ij_ is each observation of the outcome variable, μ_i_ is the global mean, treat_j_ is the main effect of the treatment, and ε_ij_ is the experimental error term. Significant gene expression differences between functional groups were accepted at *p* < 0.05, whereas significant gene expression differences between treatments were accepted at FDR (*q*-values) < 0.10.

## Results

### The Piglets' Jejunum Histomorphology

The jejunal histomorphology results (Table 2) showed that, compared to the CON group, BPC tended to increase the VH (*p* = 0.072) and VH:CD ratio (*p* = 0.060) in neonate piglets. The GC density improved in the BPC group (*p* = 0.033). However, no significant differences (*p* > 0.05) were observed in CD or IEL density.

**Table 2:** Effects of BPC supplementation during gestation of hyperprolific sows on jejunal histomorphology of neonate piglets^a^.

**Jejunum morphology**	**Treatments**		**SEM^b^**	***p*-value^c^**
	**CON**	**BPC**		
Villus height (VH), μm	621	756	55.4	0.072
Crypt depth (CD), μm	65.4	66.2	2.12	0.746
VH:CD ratio, μm/μm	9.8	11.9	0.72	0.060
Goblet cell density/100 μm VH	0.76	1.02	0.11	0.033
Lymphocyte density/100 μm VH	0.95	0.91	0.13	0.788

a *Values are expressed as means of eight newborn pigs per treatment (n = 8). Treatments: CON, control diet; BPC, control plus blend of phytogenic compounds*.

b *Standard error of the mean. Statistical significance was assumed at (p < 0.05) while statistical tendency was assumed at p < 0.10 using the T-test*.

### The Piglets' Jejunum Gene Expression

The results for the jejunum gene expression analysis of neonate piglets are shown in Table 3. Although only six genes showed statistical differences between treatments (*p* < 0.05, *q* < 0.10), the other six genes that showed statistical differences (*p* < 0.05) between functional groups are presented. Compared to the CON, a downregulation (*q* < 0.10) was observed in the BPC treatment for the *SOD2* gene from the oxidation group and the *SLC16A1/MCT1, SLC11A2/DMT1*, and *SLC39A/ZIP4* genes from the nutrient transport group, while from the immune response and barrier function groups an upregulation (*q* < 0.10) was observed for *IFNG* and *CLDN4*, respectively. Furthermore, there were no significant differences between treatments (*p* < 0.05, *q* > 0.10) for *SLC15A1/PEPT1, ALPI, IDO1, DAO1, CCK*, and *OCLN* from nutrient transport, immune response, enzyme/hormone, and barrier function group, respectively.

**Table 3:** Effects of BPC supplementation during gestation of hyperprolific sows on related jejunal health-function mRNA relative expression of neonate piglets^a^.

**Function**	**Genes^b^**	**Treatments**		**SEM^d^**	***p*-value^e^**	***q*-value^f^ (FDR)**
		**CON**	**BPC^c^**			
Oxidation	SOD2	1.00	0.71	0.051	0.009	0.023
Nutrient transport	SLC15A1/PEPT1	1.00	0.71	0.074	0.048	0.256
	SLC16A1/MCT1	1.00	0.55	0.083	0.002	0.040
	SLC11A2/DMT1	1.00	0.58	0.070	0.004	0.018
	SLC39A4/ZIP4	1.00	0.42	0.141	0.012	0.087
Immune response	IFNG	1.00	6.18	1.393	0.003	0.043
	ALPI	1.00	0.58	0.090	0.011	0.117
	IDO1	1.00	3.43	0.634	0.031	0.155
Enzyme/hormone	DAO1	1.00	0.67	0.068	0.008	0.106
	CCK	1.00	0.66	0.082	0.032	0.238
Barrier function	CLDN4	1.00	3.15	0.601	0.010	0.087
	OCLN	1.00	0.82	0.046	0.040	0.250

a *Data are means of eight newborn pigs per treatment (n = 8)*.

b *Genes: SOD2, superoxide dismutase-2; SLC15A1/PEPT1, solute carrier family 15 (oligopeptide transporter) member 1; SLC16A1/MCT1, solute carrier family 16 (monocarboxylate transporter 1) member 1; SLC11A2/DMT1, solute carrier family 11 (proton-coupled divalent metal ion transporter) member 2; SLC39A4/ZIP4, solute carrier family 39 (zinc transporter) member 4; IFNG, interferon gamma; ALPI, intestinal alkaline phosphatase; IDO1, indoleamine 2,3 dioxygenase; DAO1, diamine oxidase; CCK, cholecystokinin; CLDN4, claudin-4; OCLN, occludin. Treatments: CON, control diet; BPC, control plus blend of phytogenic compounds*.

c *Gene expression means for BPC are calculated in relation to the mean from CON group*.

d *Standard error of the mean*.

e *p-Values come from the ANOVA test*.

f *Significant gene expression differences between treatments were accepted at FDR (q-values) < 0.10*.

## Discussion

Although the study did not aim to research the sow's reproductive performance, our findings indicate that BPC improved both the total number of piglets born (19.9 vs. 17.8) and the number born alive (17.2 vs. 14.7), while the neonate piglet BW tended to decrease (1.17 vs. 1.33 kg) (Supplementary Table 1) in agreement with previous studies with a larger sample size in commercial conditions (9). LBW in piglets has been associated with a certain degree of IUGR (1), with impaired intestinal structure, and with a transcriptomic profile and bacterial colonization in neonatal IUGR piglets (13). In previous studies by our group, it has been observed that at birth, the jejunum expression of 10 genes, involved in the immune, digestive, and stress responses, is clearly different according to the piglet BW category between half-sibling piglets (light vs. average littermates) (unpublished data).

In this study we explored the effects of prenatal BPC exposure on histomorphology, and gene expression related to the health and functioning of the newborn piglet jejunum from hyperprolific dam-line sows. The jejunum plays an important role in nutrient uptake as well as immune system programming and metabolic programming in the pigs' early life (14). To our knowledge, there are no studies reporting the effects of PCs on the intestinal gene expression and morphology of neonate pigs. Positive findings were observed in histomorphology in neonate piglets of the BPC group, which showed improvements in the VH, the ratio of VH:CD, and the goblet cell density compared to newborn piglets from the CON group. The effects promoted by BPC appear to attenuate the early effects derived from IUGR, which is known to affect the intestinal cell proliferation–apoptosis balance in neonate piglets (5). They also affect the intestinal growth and morphology in association with the altered gene expression of growth-related proteins (7) and modify the volume density and function of epithelial cell types such as goblet cells, which are important constituents of the innate defense system (15). An increased VH and ratio VH:CD may suggest an improvement in the digestive and absorptive function of the intestine as a result of an increased absorptive surface, an expression of brush border enzymes, and nutrient transport systems (16).

In highly prolific sows, uterine capacity or insufficiency can affect fetal growth when competition by littermates for limited uterine space and nutrients becomes increasingly critical (17). Evidence in human studies has shown that placental uptake and transport of nutrients such as thiamin, folic acid, and glucose by BeWo cells are modulated by PCs, such as polyphenols (18). The solute carrier (SLC) genes are a large family of protein transporters in mammals, and their gene expression is notably affected if there is a deprivation of nutrients such as amino acids (AAs), which implies an effect on the capacity of the SLC to regulate intracellular nutrient concentrations and, in addition, detect alterations in extracellular nutrient levels (19). For instance, the gene expression of the SLC38 family was upregulated in hypothalamic cells N25/2 of mice after AA starvation (20). Glucose deprivation increases MCT1 protein expression and their interaction in oxidative tumor cells (21). Moreover, the hypoxia induced an increase in MCT1 plasma membrane expression in glioma cells, both in *in vitro* and *in vivo* models (22). The gene expression analysis of the current study showed that prenatal exposure of the fetus to BPC led to downregulation in the jejunum of nutrient transport-related genes *SLC16A1/MCT1, SLC11A2/DMT1*, and *SLC39A/ZIP4* in newborn piglets. This may indicate that compared to BPC, potential deprivation of nutrients, especially for Fe^2+^ (23), short-chain fatty acids (24), and zinc (25), respectively, induced an adaptative upregulation of the abovementioned SLC genes in newborn piglets from the CON group.

When inflammation occurs in the gastrointestinal tract (GIT), this can result in a decrease in digestive efficiency and reduced absorption of nutrients (26). In this study, the prenatal exposure of the fetus to BPC induced an upregulation of the immune response *IFNG* and barrier function *CLDN4* genes. IFNG is critical in increasing and mediating intestinal immunity because of its role in recognizing and eliminating pathogens. IFNG can have several functions; it can exhibit its immunomodulatory effects by controlling inflammatory response, enhancing antigen processing and presentation, increasing leukocyte trafficking, inducing an antiviral state, boosting the antimicrobial functions, and affecting cellular proliferation and apoptosis (27). On the other hand, expression of Claudin proteins such as CLDN4 within the small intestine of newborn piglets plays a vital role in controlling barrier function and mucosal homeostasis (epithelial tight junctions), particularly on the apical aspect of lateral surfaces of intestinal epithelial cells where they help regulate ion and macromolecule movement across the intestinal epithelium (28). It has been described that transcription levels of IFNG and CLDN4 showed a lower expression in LBW than normal birth weight (NBW) piglets (29). Thus, results shown in the BPC group suggest that upregulation of IFNG and CLDN4, together with the development in jejunal histomorphology, could help to improve the gut health of LBW piglets.

In addition, the induction of mitochondrial oxidative stress during periods of nutrient deprivation in animals has been associated with decreased metabolic requirements, higher mitochondrial membrane potential, and increased superoxide production at the level of the complex III of the electron transport chain (30). Since SOD2 activity is regulated in response to mitochondrial superoxide production, the upregulation of mitochondrial SOD activity results in an overproduction of hydrogen peroxide as a product of the disproportionation reaction of superoxide anion catalyzed by SOD (31). In the present study, the *SOD2* gene was downregulated in the jejunum of BPC newborn piglets, which may indicate that compared with CON, undue intrauterine oxidative stress was avoided in newborn piglets from the BPC group. In swine, it has been described that oxidative damage to DNA (8-hydroxy-20-deoxyguanosine, 8-OHdG) and proteins (carbonyls) measured in plasma increased after the stress period such as weaning, and this coincides with a rise in enzymatic antioxidant activity including SOD. Furthermore, oxidative damage to macromolecules by mitochondrial dysfunction and cellular oxidative stress is more important in LBW than in NBW piglets, as measured concentrations of 8-OHdG and carbonyls are significantly higher (32). Thus, it suggests that upregulation of the oxidation *SOD2* gene shown in CON may indicate a defense mechanism against mitochondrial dysfunction and cellular oxidative stress conditions.

It has also been reported that several dietary plants and their bioactive components have the potential to modulate human transcriptome profiles of various biological pathways involved in oxidative, immune response, glucose and fatty acid metabolism, and inflammatory and cell signaling pathways (33). In previous studies, we have observed that perinatal exposure to a blend of PCs containing eucalyptol, p-cymene, linalool, anethole, and thymol influenced the overexpression of the barrier function (MUC2), and immune response (PPARGC-α, TNF-α, TGF-β1, IDO1, and IL-10) genes in suckling piglets (9). In this study, we have observed that these effects may be anticipated by administering BPC in the sow gestation diet. In terms of fetal programming, maternal nutrition status during fetal development may result in a permanent imprint by changing the epigenetic state of the fetal genome and its gene expression (34). Although the action modes of several PCs are not yet fully clarified, the main action modes of the major compounds used in BPC, such as trans-anethole (35), 1.8-cineole (36), and Camphor (37), exhibit important biological properties. These include anti-inflammatory, antioxidant, and antimicrobial properties that could have influenced the changes on the intestinal histomorphology and gene expression of neonate piglets in this study. Therefore, the prenatal exposure of the fetus to PCs may indicate a positive means to attenuate the intestinal dysfunction in LBW piglets, especially in terms of structure and functionality.

In conclusion, BPC supplemented to hyperprolific sows during gestation influenced the prenatal programming of some intestinal biological functions. This was evidenced by the improved histomorphology and gene expression related to the nutrient absorption, immune response, intestinal integrity, and oxidative stress in the jejunum of neonate piglets.

## Data Availability Statement

The raw data supporting the conclusions of this article will be made available by the authors, without undue reservation.

## Ethics Statement

The animal study was reviewed and approved by CEEAH-Univesritat Autònoma de Barcelona.

## Author Contributions

DR-C performed the animal trial, the statistical analyses, and writing—original draft preparation. JP and DS-O were the principal investigator and contributed to conceptualization and experimental design. EP and JV conducted the conceptualization and experimental design. TA supervised the interpretation of the study results and reviewed the draft. LC-M and JF contributed to the design and set-up of the gene expression studies. All authors contributed to the article and approved the submitted version.

## Conflict of Interest

EV, TA, and JV are employees of DELACON Biotechnik GmbH. (Engerwitzdorf, Austria) which is a global supplier of phytogenic compounds. The remaining authors declare that the research was conducted in the absence of any commercial or financial relationships that could be construed as a potential conflict of interest.

## Abbreviations

BPC: blend of phytogenic compounds
GIT: gastrointestinal tract
IUGR: intrauterine growth retardation
LWB: low birth weight
NBW: normal birth weight
PCs: phytogenic compounds
VH: Villus height.
