# Supplementary material for: Phytogenic Compounds Supplemented to Gestating Hyperprolific Sows Affects the Gut Health-Related Gene Expression and Histological Responses in Neonate Piglets
Source: Front Vet Sci. 2021 Jun 14;8:639719. doi: 10.3389/fvets.2021.639719 (PMC8237712; doi:10.3389/fvets.2021.639719)
Supplement: Supplementary file 1 [file Table_1.docx]

| Supplementary Table 1. Effects of BPC dietary supplementation during gestation of hyperprolific sows on reproductive performance^a^. | | | | |
| --- | --- | --- | --- | --- |
| Item | Treatments | | SEM^c^ | *p*-value^d^ |
|  | CON | BPC |  |  |
| Parity, n | 3.42 | 3.28 | 0.624 | 0.868 |
| Sow BW, kg |  |  |  |  |
| Breeding (day 0) | 218 | 213 | 13.06 | 0.722 |
| Farrowing standardized^b^ | 266 | 263 | 10.32 | 0.844 |
| Sow reproductive performance |  |  |  |  |
| Litter birth weight, kg | 22.5 | 22.1 | 1.596 | 0.881 |
| Total born piglets, n | 17.8 | 19.9 | 0.936 | 0.089 |
| Piglets born alive, n | 14.7 | 17.2 | 0.957 | 0.040 |
| Born alive piglet BW, kg | 1.33 | 1.17 | 0.073 | 0.094 |
| ^a^Data are means of 14 sow per treatment (*n* = 14). ^b^Sow BW at day 110 of gestation less litter birth weight. Treatments: CON: control diet; BPC: control plus blend of phytogenic compounds. ^c^Standard error of the mean. ^d^Statistical significance was assumed at (*p* < 0.05) while statistical tendency was assumed at (*p* < 0.10) using T-test. | | | | |
